# CT morphological features and histogram parameters to predict micropapillary or solid components in stage IA lung adenocarcinoma

**DOI:** 10.3389/fonc.2024.1448333

**Published:** 2024-07-24

**Authors:** Qin Chen, Kaihe Lin, Baoteng Zhang, Youqin Jiang, Suying Wu, Jiajun Lin

**Affiliations:** ^1^ Department of Radiology, The First Hospital of Putian City, Putian, Fujian, China; ^2^ Department of Pathology, The First Hospital of Putian City, Putian, Fujian, China

**Keywords:** lung adenocarcinoma, CT histogram, micropapillary components, solid components, prediction model, artificial intelligence

## Abstract

**Objectives:**

This study aimed to construct prediction models based on computerized tomography (CT) signs, histogram and morphology features for the diagnosis of micropapillary or solid (MIP/SOL) components of stage IA lung adenocarcinoma (LUAC) and to evaluate the models’ performance.

**Methods:**

This clinical retrospective study included image data of 376 patients with stage IA LUAC based on postoperative pathology, admitted to Putian First Hospital from January 2019 to June 2023. According to the presence of MIP/SOL components in postoperative pathology, patients were divided into MIP/SOL+ and MIP/SOL- groups. Cases with tumors ≤ 3 cm and ≤ 2 cm were separately analyzed. Each subgroup of patients was then randomly divided into a training set and a test set in a ratio of 7:3. The training set was used to build the prediction model, and the test set was used for internal validation.

**Results:**

For tumors ≤ 3 cm, ground-glass opacity (GGO) [odds ratio (OR) = 0.244; 95% confidence interval (CI): 0.103–0.569; *p* = 0.001], entropy (OR = 1.748; 95% CI: 1.213–2.577; *p* = 0.004), average CT value (OR = 1.002; 95% CI: 1.000–1.004; *p* = 0.002), and kurtosis (OR = 1.240; 95% CI: 1.023–1.513; *p* = 0.030) were independent predictors of MIP/SOL components of stage IA LUAC. The area under the ROC curve (AUC) of the nomogram prediction model for predicting MIP/SOL components was 0.816 (95% CI: 0.756–0.877) in the training set and 0.789 (95% CI: 0.689–0.889) in the test set. In contrast, for tumors ≤ 2 cm, kurtosis was no longer an independent predictor. The nomogram prediction model had an AUC of 0.811 (95% CI: 0.731–0.891) in the training set and 0.833 (95% CI: 0.733–0.932) in the test set.

**Conclusion:**

For tumors ≤ 3 cm and ≤ 2 cm, GGO, average CT value, and entropy were the same independent influencing factors in predicting MIP/SOL components of stage IA LUAC. The nomogram prediction models have potential diagnostic value for identifying MIP/SOL components of early-stage LUAC.

## Introduction

Lung cancer is currently the leading cause of cancer-related deaths, with adenocarcinoma (AC) being the most common pathological type (1, 2). In 2011, the International Association for the Study of Lung Cancer, the American Thoracic Society, and the European Respiratory Society (IASLC/ATS/ERS) released a new classification for lung cancer, categorizing invasive lung adenocarcinoma (LUAC) into five types based on their primary pathological components: lepidic, acinar, papillary, solid (SOL), and micropapillary (MIP) (3). Significant differences in invasiveness and prognosis exist among different pathological subtypes (4, 5). Lepidic predominant AC is associated with a favorable prognosis, while SOL and MIP predominant AC have poorer prognoses (6). However, previous research has primarily focused on the relationship between the predominant pathological subtype and prognosis, overlooking the impact of minor high-risk pathological components on prognosis. An increasing number of studies have pointed out that the presence of non-dominant MIP and SOL components in early-stage LUAC is also associated with poor prognosis and early recurrence (7, 8).

Surgical resection is the most effective treatment for stage IA LUAC, including lobectomy and sub lobar resection (9). Existing studies indicate that for peripheral non-small cell lung cancer ≤ 2 cm in diameter, sublobar resection may achieve perioperative outcomes comparable to lobectomy, but patient selection must be cautious (10, 11). Previous studies have shown that sublobar resection is associated with a favorable prognosis for lepidic predominant AC (12). In contrast, lobectomy and systematic lymph node dissection are often considered for SOL and MIP predominant ACs due to their relatively higher rates of lymph node metastasis and tumor recurrence (13, 14). This underscores the importance of identifying the presence of MIP/SOL components in early-stage LUAC for patient’s surgical approach selection and prognosis assessment.

Although percutaneous biopsy can detect the pathological subtypes of LUAC preoperatively, it may increase the risk of metastasis (15). Previous studies have demonstrated the correlation between CT images and pathology, as well as prognosis (16, 17). However, traditional methods of reviewing images mainly rely on morphological features, resulting in generally limited diagnostic efficacy (18). In recent years, the rapid development of artificial intelligence in the medical field has provided a new approach to solving this problem. Computer-aided diagnostic systems utilize CT histogram technology to analyze the grayscale distribution information of the entire image, thereby improving the accuracy and sensitivity of diagnosis (19). However, the effectiveness of CT histogram parameters to predict MIP/SOL components of stage IA LUAC still needs validation. This study aims to construct nomogram prediction models by combining preoperative CT morphological features and histogram parameters to explore their value in predicting the presence of MIP/SOL structures in stage IA LUAC.

## Materials and methods

### Study population

**Figure 1:**
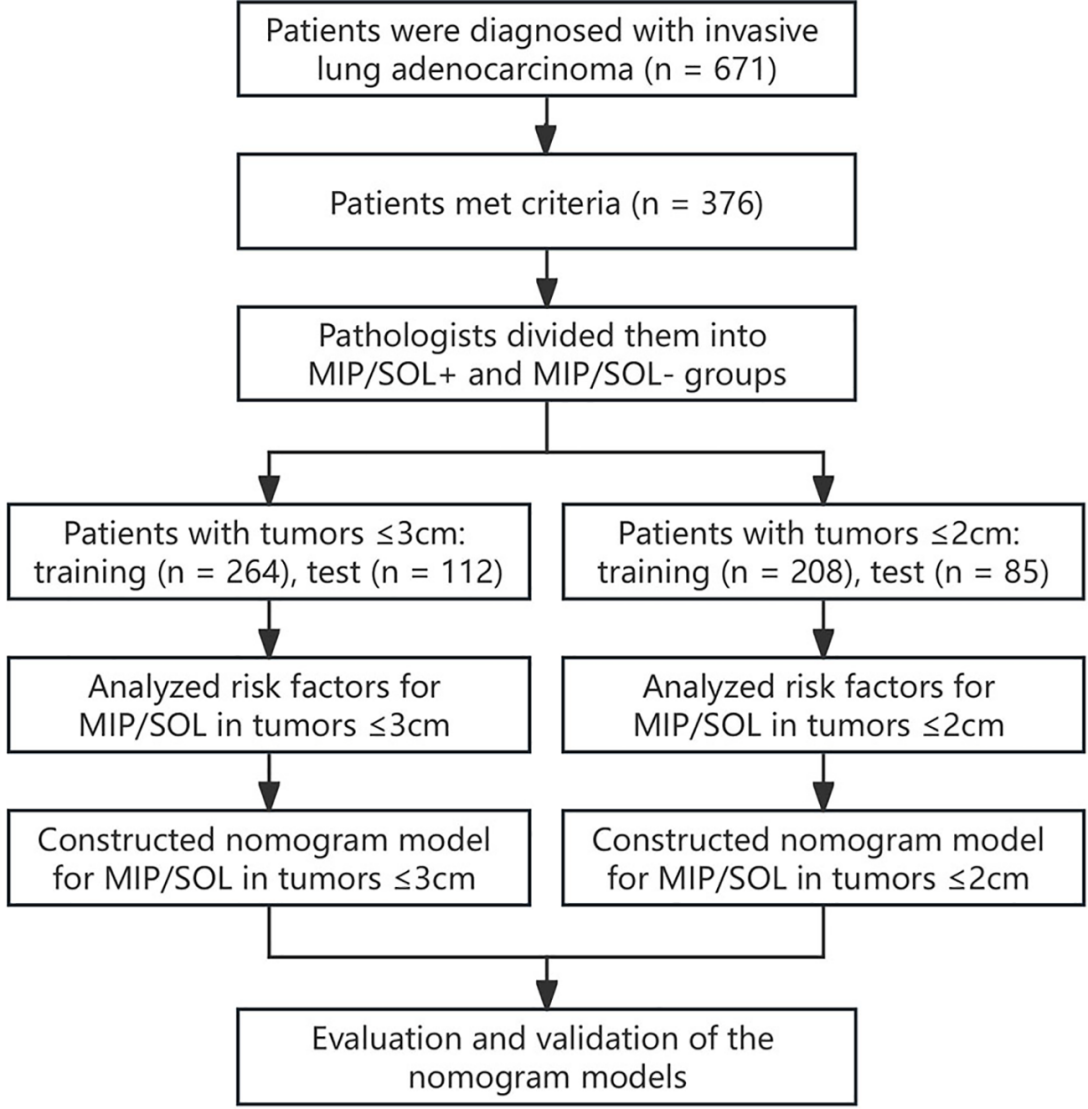
Research flow chart.

This retrospective study included patients diagnosed with stage IA invasive LUAC who underwent thin-section CT scans of the lungs and subsequent surgical pathology confirmation at the First Hospital of Putian City from January 2019 to June 2023. The clinical information and image data were recorded. The inclusion criteria were as follows: patients who 1) were diagnosed with stage IA LUAC confirmed by surgical pathology, with a lesion longest diameter ≤ 3cm; 2) had clear thin-section CT lung images that a computer-aided diagnosis system could accurately identify the lesions; and 3) had complete clinical data and laboratory examination results. The exclusion criteria were: Patients who 1) received prior radiotherapy, chemotherapy, targeted therapy, immunotherapy, or radiofrequency ablation before surgery; 2) had an interval between preoperative examination and surgery exceeding one month; 3) had pathological types including mucinous AC, enteric AC, colloid AC, and fetal AC; and 4) had multiple lung nodules containing both MIP/SOL+ and MIP/SOL- nodules. This retrospective study has been approved by the hospital’s ethics committee (approval number 2023-068). The specific workflow is illustrated in **Figure 1**.

### Chest CT examination and image analysis

The chest thin-section scans were performed using Siemens DECT or Philips 64-slice spiral CT scanners. Patients were placed in a supine position, and volumetric data were collected at the end of a deep inspiration with breath-holding. All parameters were derived from the CT plain images. The scan range extended from the lung apices to the adrenal glands. The scan parameters were set as follows: tube voltage ranged from 100 to 120 kV, tube current was automatically adjusted, matrix size was 512×512, slice thickness during acquisition was 5.00 mm, and the reconstructed image slice thickness was either 1.25 mm or 1.00 mm.

The morphological features of CT images were evaluated by two experienced radiologists (with 10 and 11 years of experience, respectively) in a double-blinded manner. Disagreements were resolved through discussion. Morphological features included: 1) Location: divided into left upper lobe, left lower lobe, right upper lobe, right middle lobe, and right lower lobe; 2) Nodule: categorized as ground glass opacity (GGO)-containing nodules (pure GGO nodules and part-solid nodules) or solid nodules. GGO refers to areas in the lungs that appear hazy or translucent on high-resolution CT scans and do not obscure the underlying bronchial structures or pulmonary blood vessels; 3) Lobulation sign: uneven, scalloped edges around the nodule; 4) Spiculation sign: spiky or linear projections extending from the nodule’s edge; 5) Vacuole sign: the presence of small air lucencies within the nodule; 6) Bronchial inflation sign: presence of air-filled bronchial structures within the nodule; 7) Vascular convergence sign: blood vessels converging towards the nodule; 8) Pleural indentation: indentation of the pleura or interlobar fissures near the nodule.

Subsequently, images were imported into a computer-aided diagnostic system (Chest-Lung: 3.3.1, developed by Shukun Network Technology Co., Ltd.), which automatically identified and analyzed nodules’ quantitative histogram features. One radiologist collected the following data: 1) CT values: maximum, minimum, average, median, and standard deviation; 2) Grayscale histogram features: kurtosis (reflects the steepness of the gray value of CT images), skewness (reflects the asymmetry of the grayscale distribution in CT images), and entropy (describes the chaos of the image grayscale distribution in CT images); 3) Geometric features: compactness (describes how tightly the nodule’s shape is packed), sphericity (indicates how spherical the nodule is), and max slice area (measures the largest cross-sectional area of the nodule); 4) Consolidation-to-tumor ratio (CTR): calculates the proportion of the solid component within the entire nodule; 5) Energy: reflects the uniformity and frequency characteristics of the grayscale distribution; 6) 3D long axis (mm); 7) Total nodule volume (mm^3^); 8) Nodule mass (mg). The other radiologist verified whether the system correctly identified the nodules’ range.

### Clinical data and serum tumor markers

This study collected demographic characteristics (age, sex, smoking), history of systemic tumors, family history of lung cancer, and laboratory indicators (carcinoembryonic antigen [CEA], cytokeratin 19 fragment [CYFRA21-1], neuron-specific enolase [NSE], and pro-gastrin-releasing peptide [ProGRP]). The reference ranges for tumor markers were defined as CEA: 0–5.00 ng/mL; CYFRA21-1: 0–3.60 ng/mL; NSE: 0–15.20 ng/mL; ProGRP: 0–65 pg/mL.

### Pathological histological classification

According to the 8th edition of the TNM staging standard revised by the International Union Against Cancer, cases of stage IA invasive LUAC were included. For stage IA, including T_1a_N_0_M_0_, T_1b_N_0_M_0_, and T_1c_N_0_M_0_; T_1a_ indicates a tumor ≤ 1 cm in greatest dimension, T_1b_ refers to a tumor > 1 cm but ≤ 2 cm, and T_1c_ represents a tumor > 2 cm but ≤ 3 cm. N_0_ indicates no regional lymph node metastasis, and M_0_ indicates no distant metastasis (20). The pathological subtype classification of all cases of LUAC was determined by intermediate-level pathologists in the pathology department using the 5th edition of the WHO classification of thoracic tumors proposed in 2021. The specific subtype classification was described in increments of 5%, including lepidic, acinar, papillary, micropapillary, and solid types (21). We defined stage IA LUAC with ≥ 5% micropapillary and/or ≥ 5% solid components as MIP/SOL+, while the remaining cases were classified as MIP/SOL-.

### Statistical analysis

Data analysis was performed using SPSS version 26.0 and R version 4.1.2 statistical software. Normally distributed or approximately normally distributed metric data were presented as mean ± standard deviation (SD) and compared by independent sample *t*-tests. Skewed metric data were presented as median [interquartile range (IQR)], and compared by Mann-Whitney U tests. Count data were presented as frequency and percentage (%) and compared using Pearson’s chi-square test, continuity corrected chi-square test, or Fisher’s exact test. The data were randomly divided into a training set and a test set in a 7:3 ratio using the sample function in R software. LASSO regression was used to select variables in the training set. Logistic regression analysis was performed to select independent predictive factors for MIP/SOL components of stage IA LUAC and construct the models. Nomograms were used to visualize the models. The receiver operating characteristic curve (ROC) and area under curve (AUC) were used to examine the models’ performance. Calibration curves and Hosmer-Lemeshow goodness-of-fit test were applied to evaluate calibration. Decision curve analysis (DCA) and clinical impact curves (CIC) were performed to evaluate the clinical effectiveness of the models. A significance level of *p* < 0.05 was considered statistically significant.

## Results

### Baseline characteristics

This study included a total of 376 patients with stage IA invasive LUAC (149 males and 227 females, aged 33-84 years old). The data were randomly divided into a training set (n = 264) and a test set (n = 112) in a 7:3 ratio. All indicators between the two datasets were comparable (*p* > 0.05) (**Supplementary Table S1**).

### Independent risk factors for MIP/SOL components in stage IA LUAC with tumors ≤ 3cm

**Figure 2:**
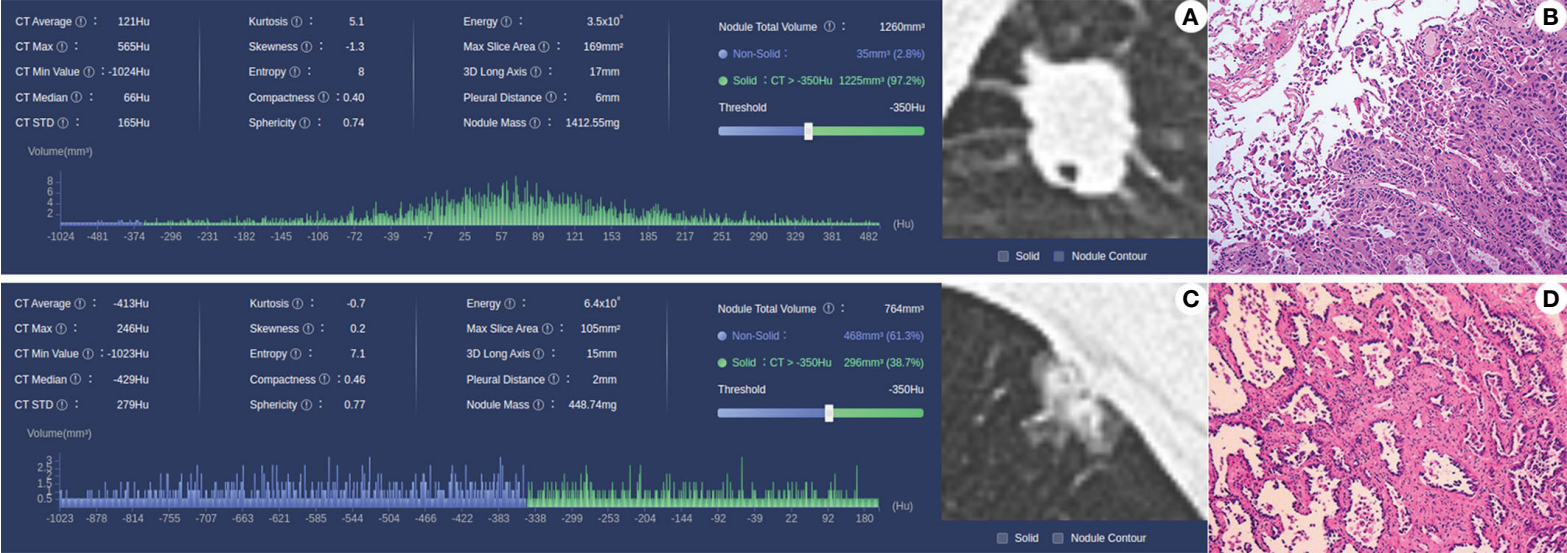
**(A, B)** Case 1, male, 56 years old, MIP/SOL+ group lung AC, irregular solid nodule in the middle lobe of the right lung, with a maximum diameter of about 18 mm, with lobulated and spiculated edges and vacuole signs within the lesion; pathology (HE × 10) shows invasive lung AC, with micropapillary accounting for approximately 50% and acinar type accounting for approximately 50%. **(C, D)** Case 2: Male, 63 years old, lung AC in the MIP/SOL- group; mixed ground-glass nodule in the upper lobe of the left lung, with a maximum diameter of about 15 mm, lobulated edges, and adjacent pleural depression; pathology (HE × 10) showed invasive lung AC, with approximately 40% lepidic structures and 60% acinar type.

In the training set, patients were divided into MIP/SOL+ and MIP/SOL- groups based on the presence or absence of micropapillary/solid components. There were 202 cases in the MIP/SOL- group (71 males, median age of 62 [IQR: 53, 67]) and 62 cases in the MIP/SOL+ group (31 males, median age of 61 [IQR: 53, 66]). Clinical data showed statistically significant differences in sex and CEA levels between the two groups (both *p* < 0.05). There were no statistically significant differences in age, smoking history, family history of lung cancer, presence of cavitation, NSE, CYFRA21-1, and ProGRP levels between the groups (all *p* > 0.05), as detailed in **Table 1**. Typical cases from both groups are illustrated in **Figure 2**.

**Table 1:** Comparison of clinical data indicators between the two groups in the training set for tumors ≤ 3cm.

	MIP/SOL- (n=202)	MIP/SOL+ (n=62)	*P* value
Age, years old	62.0 [53.0, 67.0]	61.0 [53.0, 66.0]	0.757^*^
Sex, n (%)			0.036^#^
Female	71 (35.1)	31 (50.0)	
Male	131 (64.9)	31 (50.0)	
History of smoking, n (%)			0.752^#^
No	182 (90.1)	55 (88.7)	
Yes	20 (9.9)	7 (11.3)	
Family history of lung cancer, n (%)			0.897^△^
No	196 (97.0)	61 (98.4)	
Yes	6 (3.0)	1(1.6)	
CEA, n (%)			0.037^#^
Normal	185 (91.6)	51 (82.3)	
Rise	17 (8.4)	11 (17.7)	
CYFRA21-1, n (%)			0.918^#^
Normal	165 (81.7)	51 (82.3)	
Rise	37 (18.3)	11 (17.7)	
NSE, n (%)			0.349^#^
Normal	143 (70.8)	40 (64.5)	
Rise	59 (29.2)	22 (35.5)	
ProGRP, n (%)			0.100^#^
Normal	189 (93.6)	54 (87.1)	
Rise	13 (6.4)	8 (12.9)	

CEA,Carcinoembryonic antigen; CYFRA21-1,Cytokeratin 19; MIP, micropapillary; NSE,Neuron specific enolase; ProGRP, Pro-gastrin-releasing peptide; SOL,solid.

# Pearsonχ^2^ test; △ continuous adjusted χ^2^ test; * Mann-Whitney U test.

**Figure 3:**
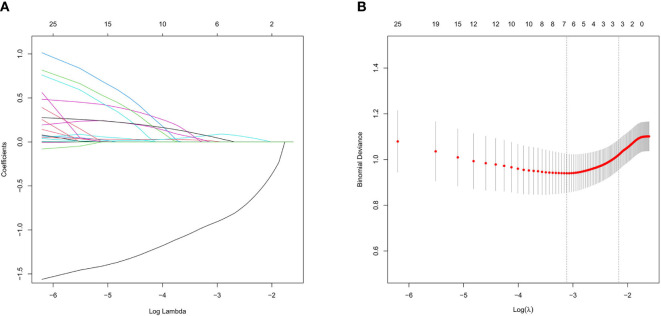
Lasso regression analysis diagram in the training set for tumors ≤ 3cm. **(A)** Coefficient path of Lasso regression. **(B)** Lasso regression cross-validation results.

Screening of CT morphological features and histogram parameters using LASSO regression (**Figure 3**). Lasso regression with five-fold cross-validation was used to determine the optimal penalty coefficient λ. In **Figure 3B**, lines were drawn at λ (0.044) and λ+SE (0.115), respectively. Then, λ (0.044) was considered as a benchmark to select six factors with non-zero regression coefficients as potential predictive factors for MIP/SOL components of stage IA LUAC, including GGO, average CT value, 3D long axis, energy, entropy, and kurtosis. The above meaningful clinical and CT characteristics were included in binary multifactor logistic regression analysis, and the backward stepwise regression method was used to finally screen out four independent predictive factors (all *p* < 0.05), including GGO, average CT value, entropy, and kurtosis (**Table 2**).

**Table 2:** Multivariate logistic regression analysis for predicting MIP/SOL components in tumors ≤ 3cm.

Variable	*OR*	95%*CI*	*P* value
GGO	0.244	0.103-0.569	0.001
Entropy	1.748	1.213-2.577	0.004
Kurtosis	1.240	1.023-1.513	0.030
AverageCTvalue	1.002	1.000-1.004	0.002

GGO, ground-glass opacity; CT, computed tomography.

### Nomogram construction, evaluation, and validation for MIP/SOL components in LUAC with tumors ≤ 3cm

**Figure 4:**
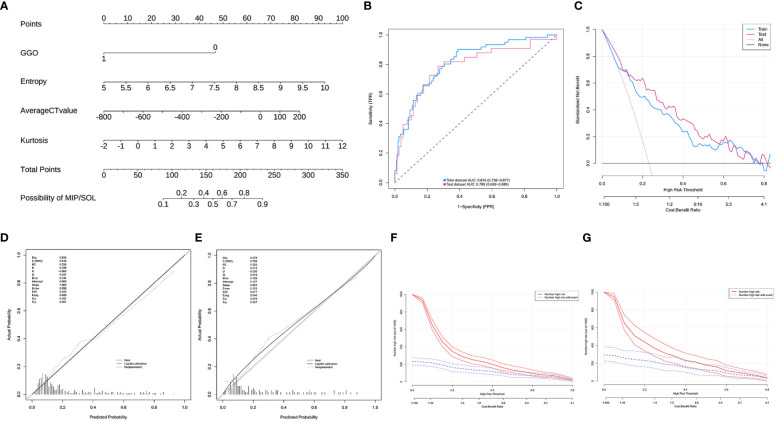
**(A)** Model 1, nomogram prediction model for the risk of MIP/SOL in stage IA lung AC with tumors ≤ 3cm. **(B)** Training set and test set ROC curves. **(C)** Training set and test set DCA curves, showing that when the threshold probabilities of the training set and test set are 0.08–0.78 and 0.12–0.78, respectively, the patient’s net benefit rate is greater than 0. **(D,E)** Training set and test set calibration curves. **(F,G)** Training set and test set CIC curves, indicating that for risk thresholds > 0.40, the model’s predictions align closely with the actual high-risk MIP/SOL population. GGO, ground-glass opacity; CT, computed tomography; MIP, micropapillary; SOL, solid.

A nomogram prediction model was constructed based on the results of the multifactor logistic regression analysis. The AUC of the nomogram prediction model in the training set was 0.816 (95% CI: 0.756–0.877), the sensitivity was 0.902, and the specificity was 0.611. The AUC in the validation set was 0.789 (95%CI: 0.689–0.889), the sensitivity was 0.818, and the specificity was 0.596. It indicates that the prediction model has decent discriminatory ability. The calibration curves of the training set and test set show that the predicted probability of the model is close to the actual probability, and the calibration is good. The Hosmer-Lemeshow test results show that in the training set χ^2^ = 9.785, *p* = 0.280; in the test set χ^2^ = 3.898, *p* = 0.866, indicating that the goodness of fit of the model is good. The DCA and CIC results demonstrated the nomogram prediction model’s decent clinical applicability (**Figure 4**).

### Construction, evaluation, and validation of prediction models for MIP/SOL components in LUAC with tumors ≤ 2cm

**Figure 5:**
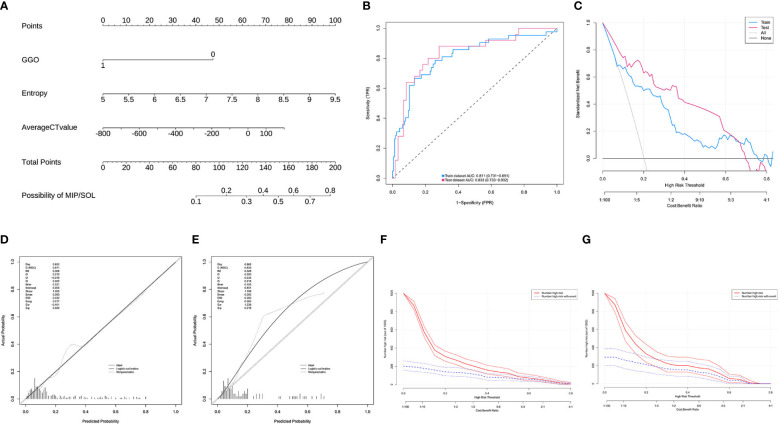
**(A)** Model 2, nomogram prediction model for the risk of MIP/SOL in stage IA lung AC with tumors ≤ 2cm. **(B)** ROC curves for the training and test sets. **(C)** DCA curves for the training and test sets, showing that when the threshold probabilities of the training set and test set were 0.08–0.76 and 0.01–0.70, respectively, the patient’s net benefit rate was greater than 0. **(D, E)** Calibration curves for the training and test sets. **(F, G)** CIC curves for the training and test sets, showing that for risk thresholds > 0.30, the model’s predictions correspond well with the actual high-risk MIP/SOL population. GGO, ground-glass opacity; CT, computed tomography; MIP, micropapillary; SOL, solid.

In this study, a total of 293 patients with stage IA invasive LUAC with tumor diameters ≤ 2cm were included. The data were randomly divided into a training set (n = 208) and a test set (n = 85) in a 7:3 ratio. All indicators between the two datasets were comparable (*p* > 0.05) (**Supplementary Table S2**). There were no statistically significant differences in clinical data indicators in the training set (*p* > 0.05) (**Supplementary Table S3**). LASSO regression was used to screen CT histogram features and morphological features (**Supplementary Figure S1**). Finally, λ (0.050) was used as the standard to select five factors with non-zero regression coefficients, including GGO, location, average CT value, entropy, and median CT value. Subsequently, through backward stepwise regression analysis of binary multifactor logistic regression, three independent predictive factors (all *p* < 0.05) were finally selected, including GGO, average CT value, and entropy (**Supplementary Table S4**). The nomogram prediction model based on multifactor logistic regression analysis was constructed. The AUC in the training set was 0.811 (95% CI: 0.731–0.891), the sensitivity was 0.667, and the specificity was 0.867. The AUC in the validation set was 0.833 (95% CI: 0.733–0.932), the sensitivity was 0.640, and the specificity was 0.917. Calibration curves and Hosmer-Lemeshow test results showed that the model had good calibration. The DCA and CIC curves indicated that the nomogram prediction model has decent clinical applicability (**Figure 5**).

## Discussion

Surgical resection is the most effective treatment for stage IA LUAC; however, postoperative recurrence remains a concern (9, 22). Existing studies indicate that high-risk pathological features such as MIP/SOL components, spread through air spaces, lymphovascular invasion, and visceral pleural invasion in stage IA LUAC significantly impact prognosis, making early and accurate identification crucial for optimizing clinical decision-making (23–25). MIP/SOL components are more invasive compared to other subtypes like lepidic, acinar, and papillary (6). They are independent predictors of postoperative recurrence in stage IA LUAC (26). However, there is currently no reliable non-invasive method to accurately identify MIP/SOL components preoperatively. Previous studies have demonstrated the correlation between CT images and pathology, as well as prognosis (16, 17). For instance, Kim et al. pointed out that spiculation in CT images is associated with higher recurrence rates and poorer survival rates (16). Similarly, Cai et al. found that GGO components in CT images are related to the lepidic predominant subtype of lung adenocarcinoma (27). Additionally, CT histograms can extract features from CT images that are difficult for the human eye to detect, thereby improving predictive accuracy (19). This study aims to establish predictive models by combining CT morphological features and histogram parameters to help clinicians preoperatively identify MIP/SOL components. The results showed that, for LUACs ≤ 3 cm or ≤ 2 cm, the nomogram models effectively predict MIP/SOL components. Among the predictive factors, GGO, average CT value, and entropy were consistently important.

This study found that stage IA LUACs ≤ 3 cm and ≤ 2 cm containing GGO were less likely to exhibit MIP/SOL components (OR = 0.244, *p* = 0.001), consistent with the findings of Katsumata et al., who also noted that lesions with GGO are more likely to be low-risk (28). Additionally, a validation study based on data from the Japanese Clinical Oncology Group study JCOG0201 by Hattori et al. found that ground-glass nodules (pure ground-glass nodules and partially solid nodules) had significantly higher 5-year overall survival rates compared to solid nodules (95.1% vs. 81.1%), regardless of the size of the solid component (29). The formation of GGO is likely related to mild infiltration of tumor cells into the alveolar wall, local fibrosis, and scar formation, characteristics associated with low-grade malignancy, slower growth, and lower cellular proliferation activity (27, 30). In contrast, MIP and SOL components represent more invasive and malignant subtypes, with active cell proliferation and rapid growth, leading to the formation of solid portions. Therefore, GGOs are less likely to contain MIP/SOL components, highlighting the biological and cytological differences between these pathological subtypes.

Additionally, average CT value and entropy were also independent predictive factors for MIP/SOL components in IA-stage LUAC in the subgroups of ≤ 3 cm and ≤ 2 cm. The average CT value was significantly higher in the MIP/SOL+ group compared to the MIP/SOL- group (OR = 1.002, *p* = 0.002). This finding is consistent with the notion that the average CT value reflects the overall density of the lesion, with MIP/SOL components more likely to appear as solid nodules on CT images. Yoshida et al.’s study supports this, showing that MIP components are more common in solid nodules than in pure GGOs or subsolid nodules (31). A meta-analysis further confirmed that the average CT value has good diagnostic performance in predicting the invasiveness of GGOs (32). Additionally, entropy measures the disorder of the grayscale distribution in CT images, with higher values indicating more chaotic and irregular grayscale distributions. This study found that higher entropy values were associated with a higher risk of MIP/SOL components (OR = 1.748, *p* = 0.004). Qiu et al. also identified entropy as an independent predictor for quantifying the invasiveness of stage IA LUAC using CT texture features (33). This may be due to the higher malignancy of MIP/SOL components, leading to increased tissue heterogeneity and grayscale irregularity in the tumor, resulting in higher entropy values (34).

It is worth noting that the results for kurtosis were inconsistent in the subgroups of ≤ 3 cm and ≤ 2 cm. In the ≤ 3 cm subgroup, kurtosis was an important predictive factor for MIP/SOL components in stage IA LUAC (OR = 1.240, *p* = 0.030). Kurtosis describes the steepness of the grayscale value distribution in CT images, with higher values indicating steeper distributions and suggesting denser structures within the nodules. This study found that higher kurtosis was associated with an increased risk of MIP/SOL components, possibly due to the high malignancy, active cell proliferation, and rapid growth of MIP/SOL components, leading to dense cell accumulation. This finding is similar to the results of Alpert et al., who found that kurtosis has statistical significance in distinguishing between different invasive subtypes of LUAC (35). However, in the ≤ 2 cm subgroup analysis, kurtosis did not demonstrate the same predictive capability. We speculate that this may be because smaller tumors have less internal tissue heterogeneity and smaller differences in grayscale distribution, making kurtosis differences less apparent. As tumors grow larger, tissue heterogeneity increases, leading to greater differences in grayscale distribution and making kurtosis a significant predictive factor. The inconsistency in subgroup analysis results highlights the necessity of developing predictive models tailored to different tumor sizes. Our study constructed nomogram models for stage IA lung adenocarcinoma patients based on tumor size (≤ 3 cm and ≤ 2 cm). The ROC curve shows that the models have decent diagnostic performance.

Recent studies have highlighted the importance of predictive models in assessing the invasiveness of lung adenocarcinoma and the recurrence of low-risk resected stage I lung adenocarcinoma (36, 37). Meanwhile, researchers have developed nomogram models based on radiomic features for predicting the invasiveness of LUAC, demonstrating high sensitivity and specificity (38). However, radiomic data processing is complex, reproducibility is challenging, and clinical applicability is limited (39). The nomogram models proposed in this study, based on computer-aided diagnosis system CT histogram parameters, offers simple data acquisition and high stability (40). The DCA and CIC results demonstrated the model’s decent clinical applicability. Medical staff can use the nomogram model to preoperatively calculate the probability of MIP/SOL components in each LUAC patient, providing a basis for clinical decision-making and personalized treatment planning.

However, this study still has some limitations: first, due to the single-center research design, central bias may have been introduced; second, selection bias in retrospective analysis may exist. To strengthen the credibility of the conclusions, more rigorous designs, including multi-center and prospective cohort studies, will be used in the future to further confirm the research results. Additionally, future research will continue to explore the relationship between other high-risk pathological features and CT images in stage IA lung adenocarcinoma.

## Conclusion

In summary, the nomogram models established for lung adenocarcinomas ≤ 3 cm and ≤ 2 cm demonstrated decent accuracy and clinical applicability in predicting MIP/SOL components. GGO, average CT value, and entropy were consistent predictors for MIP/SOL components in stage IA lung adenocarcinoma smaller than 3 cm and 2 cm. The nomogram prediction model has potential diagnostic value for the non-invasive identification of MIP/SOL components in early-stage LUAC, providing a valuable tool for clinical decision-making and personalized treatment planning.

## Data availability statement

The original contributions presented in the study are included in the article/**Supplementary Material**. Further inquiries can be directed to the corresponding author.

## Ethics statement

The studies involving humans were approved by Ethics Committee of Putian First Hospital. The studies were conducted in accordance with the local legislation and institutional requirements. The ethics committee/institutional review board waived the requirement of written informed consent for participation from the participants or the participants’ legal guardians/next of kin because This is a retrospective single-center study. According to national legislation and institutional requirements, written informed consent was not required for this study.

## Author contributions

QC: Data curation, Formal analysis, Visualization, Writing – original draft. KL: Methodology, Project administration, Writing – original draft. BZ: Data curation, Software, Writing – original draft. YJ: Investigation, Resources, Visualization, Writing – original draft. SW: Project administration, Resources, Writing – review & editing. JL: Conceptualization, Supervision, Writing – review & editing.

## References

[1] Bray F Laversanne M Sung H Ferlay J Siegel RL Soerjomataram I. Global cancer statistics 2022: GLOBOCAN estimates of incidence and mortality worldwide for 36 cancers in 185 countries. CA Cancer J Clin. (2024) 74:229–63. doi: 10.3322/caac.21834 38572751

[2] Ganti AK Klein AB Cotarla I Seal B Chou E. Update of incidence, prevalence, survival, and initial treatment in patients with non-small cell lung cancer in the US. JAMA Oncol. (2021) 7:1824–32. doi: 10.1001/jamaoncol.2021.4932 PMC8532041 34673888

[3] Travis WD Brambilla E Noguchi M Nicholson AG Geisinger KR Yatabe Y. International association for the study of lung cancer/american thoracic society/european respiratory society international multidisciplinary classification of lung adenocarcinoma. J Thorac Oncol. (2011) 6:244–85. doi: 10.1097/JTO.0b013e318206a221 PMC4513953 21252716

[4] Miyahara N Nii K Benazzo A Hoda MA Iwasaki A Klepetko W. Solid predominant subtype in lung adenocarcinoma is related to poor prognosis after surgical resection: A systematic review and meta-analysis. Eur J Surg Oncol. (2019) 45:1156–62. doi: 10.1016/j.ejso.2019.01.220 30772108

[5] Kuhn E Morbini P Cancellieri A Damiani S Cavazza A Comin CE. Adenocarcinoma classification: patterns and prognosis. Pathologica. (2018) 110:5–11. 30259909

[6] Lin W Huang M Zhang Z Chai T Chen S Gao L. A retrospective study of the relationship between the pathologic subtype and lymph node metastasis of lung adenocarcinomas of ≤3 cm diameter. Med (Baltimore). (2020) 99:e21453. doi: 10.1097/MD.0000000000021453 PMC7478443 32898994

[7] Choi SH Jeong JY Lee SY Shin KM Jeong SY Park TI. Clinical implication of minimal presence of solid or micropapillary subtype in early-stage lung adenocarcinoma. Thorac Cancer. (2021) 12:235–44. doi: 10.1111/1759-7714.13754 PMC7812076 33231358

[8] Wang W Hu Z Zhao J Huang Y Rao S Yang J. Both the presence of a micropapillary component and the micropapillary predominant subtype predict poor prognosis after lung adenocarcinoma resection: a meta-analysis. J Cardiothorac Surg. (2020) 15:1–8. doi: 10.1186/s13019-020-01199-8 32600473 PMC7325156

[9] Ettinger DS Wood DE Aisner DL Akerley W Bauman JR Bharat A. NCCN guidelines insights: non-small cell lung cancer, version 2.2021. J Natl Compr Canc Netw. (2021) 19:254–66. doi: 10.6004/jnccn.2021.0013 33668021

[10] Altorki NK Wang X Wigle D Gu L Darling G Ashrafi AS. Perioperative mortality and morbidity after sublobar versus lobar resection for early-stage non-small-cell lung cancer: post-hoc analysis of an international, randomised, phase 3 trial (CALGB/Alliance 140503). Lancet Respir Med. (2018) 6:915–24. doi: 10.1016/S2213-2600(18)30411-9 PMC6396275 30442588

[11] Saji H Okada M Tsuboi M Nakajima R Suzuki K Aokage K. Segmentectomy versus lobectomy in small-sized peripheral non-small-cell lung cancer (JCOG0802/WJOG4607L): a multicentre, open-label, phase 3, randomised, controlled, non-inferiority trial. Lancet. (2022) 399:1607–17. doi: 10.1016/S0140-6736(21)02333-3 35461558

[12] Suzuki K Watanabe SI Wakabayashi M Saji H Aokage K Moriya Y. A single-arm study of sublobar resection for ground-glass opacity dominant peripheral lung cancer. J Thorac Cardiovasc Surg. (2022) 163:289–301. doi: 10.1016/j.jtcvs.2020.09.146 33487427

[13] Xu L Zhou H Wang G Huang Z Xiong R Sun X. The prognostic influence of histological subtypes of micropapillary tumors on patients with lung adenocarcinoma ≤ 2 cm. Front Oncol. (2022) 12:954317. doi: 10.3389/fonc.2022.954317 36033545 PMC9399672

[14] Chang C Sun X Zhao W Wang R Qian X Lei B. Minor components of micropapillary and solid subtypes in lung invasive adenocarcinoma (≤ 3 cm): PET/CT findings and correlations with lymph node metastasis. Radiol Med. (2020) 125:257–64. doi: 10.1007/s11547-019-01112-x 31823295

[15] Hong H Hahn S Matsuguma H Inoue M Shintani Y Honda O. Pleural recurrence after transthoracic needle lung biopsy in stage I lung cancer: a systematic review and individual patient-level meta-analysis. Thorax. (2021) 76:582–90. doi: 10.1136/thoraxjnl-2020-216492 33723018

[16] Kim H Park CM. Tumor-associated prognostic factors extractable from chest CT scans in patients with lung cancer. Transl Lung Cancer Res. (2023) 12:1133–9. doi: 10.21037/tlcr-22-904 PMC10261868 37323175

[17] Li X Zhang W Yu Y Zhang G Zhou L Wu Z. CT features and quantitative analysis of subsolid nodule lung adenocarcinoma for pathological classification prediction. BMC Cancer. (2020) 20:60. doi: 10.1186/s12885-020-6556-6 31992239 PMC6986053

[18] Dai J Yu G Yu J. Can CT imaging features of ground-glass opacity predict invasiveness? A meta-analysis. Thorac Cancer. (2018) 9:452–8. doi: 10.1111/1759-7714.12604 PMC5879054 29446528

[19] Chen J Cao R Jiao S Dong Y Wang Z Zhu H. Application value of a computer-aided diagnosis and management system for the detection of lung nodules. Quant Imaging Med Surg. (2023) 13:6929–41. doi: 10.21037/qims-22-1297 PMC10585542 37869302

[20] Goldstraw P Chansky K Crowley J Rami-Porta R Asamura H Eberhardt WEE. The IASLC lung cancer staging project: proposals for revision of the TNM stage groupings in the forthcoming (Eighth) edition of the TNM classification for lung cancer. J Thorac Oncol. (2016) 11:39–51. doi: 10.1016/j.jtho.2015.09.009 26762738

[21] Tsao MS Nicholson AG Maleszewski JJ Marx A Travis WD. Introduction to 2021 WHO classification of thoracic tumors. J Thorac Oncol. (2022) 17:e1–4. doi: 10.1016/j.jtho.2021.09.017 34930611

[22] Watanabe K Sakamaki K Ito H Yokose T Yamada K Nakayama H. Impact of the micropapillary component on the timing of recurrence in patients with resected lung adenocarcinoma. Eur J Cardio-Thoracic Surg. (2020) 58:1010–8. doi: 10.1093/ejcts/ezaa138 32386405

[23] Fick CN Dunne EG Vanstraelen S Toumbacaris N Tan KS Rocco G. High-risk features associated with recurrence in stage I lung adenocarcinoma. J Thorac Cardiovasc Surg. (2024), S0022–5223(24)00440-9. doi: 10.1016/j.jtcvs.2024.05.009 PMC11582076 38788834

[24] Toyokawa G Yamada Y Tagawa T Kamitani T Yamasaki Y Shimokawa M. Computed tomography features of resected lung adenocarcinomas with spread through air spaces. J Thorac Cardiovasc Surg. (2018) 156:1670–1676.e4. doi: 10.1016/j.jtcvs.2018.04.126 29961590

[25] Bains S Eguchi T Warth A Yeh YC Nitadori JI Woo KM. Procedure-specific risk prediction for recurrence in patients undergoing lobectomy or sublobar resection for small (≤2 cm) lung adenocarcinoma: an international cohort analysis. J Thorac Oncol. (2019) 14:72–86. doi: 10.1016/j.jtho.2018.09.008 30253972 PMC6309652

[26] Zhang L Liu J Yang D Ni Z Lu X Liu Y. A nomogram based on consolidation tumor ratio combined with solid or micropapillary patterns for postoperative recurrence in pathological stage IA lung adenocarcinoma. Diagnostics (Basel). (2023) 13:2376. doi: 10.3390/diagnostics13142376 37510119 PMC10378621

[27] Cai Y Chen T Zhang S Tan M Wang J. Correlation exploration among CT imaging, pathology and genotype of pulmonary ground-glass opacity. J Cell Mol Med. (2023) 27:2021–31. doi: 10.1111/jcmm.17797 PMC10339074 37340599

[28] Katsumata S Aokage K Ishii G Hoshino H Suzuki J Miyoshi T. Pathological features and prognostic implications of ground-glass opacity components on computed tomography for clinical stage I lung adenocarcinoma. Surg Today. (2021) 51:1188–202. doi: 10.1007/s00595-021-02235-3 33745094

[29] Hattori A Suzuki K Takamochi K Wakabayashi M Aokage K Saji H. Prognostic impact of a ground-glass opacity component in clinical stage IA non-small cell lung cancer. J Thorac Cardiovasc Surg. (2021) 161:1469–80. doi: 10.1016/j.jtcvs.2020.01.107 32451073

[30] Zhang P Li T Tao X Jin X Zhao S. HRCT features between lepidic-predominant type and other pathological subtypes in early-stage invasive pulmonary adenocarcinoma appearing as a ground-glass nodule. BMC Cancer. (2021) 21:1124. doi: 10.1186/s12885-021-08821-5 34666705 PMC8524968

[31] Yoshida Y Nitadori JI Shinozaki-Ushiku A Sato J Miyaji T Yamaguchi T. Micropapillary histological subtype in lung adenocarcinoma of 2 cm or less: impact on recurrence and clinical predictors. Gen Thorac Cardiovasc Surg. (2017) 65:273–9. doi: 10.1007/s11748-017-0747-3 28243892

[32] He S Chen C Wang Z Yu X Liu S Huang Z. The use of the mean computed-tomography value to predict the invasiveness of ground-glass nodules: A meta-analysis. Asian J Surg. (2023) 46:677–82. doi: 10.1016/j.asjsur.2022.07.031 35864044

[33] Qiu ZB Zhang C Chu XP Cai FY Yang XN Wu YL. Quantifying invasiveness of clinical stage IA lung adenocarcinoma with computed tomography texture features. J Thorac Cardiovasc Surg. (2022) 163:805–815.e3. doi: 10.1016/j.jtcvs.2020.12.092 33541730

[34] Chen X Feng B Chen Y Hao Y Duan X Cui E. Whole-lesion computed tomography-based entropy parameters for the differentiation of minimally invasive and invasive adenocarcinomas appearing as pulmonary subsolid nodules. J Comput Assist Tomogr. (2019) 43:817–24. doi: 10.1097/RCT.0000000000000889 31343995

[35] Alpert JB Rusinek H Ko JP Dane B Pass HI Crawford BK. Lepidic predominant pulmonary lesions (LPL): CT-based distinction from more invasive adenocarcinomas using 3D volumetric density and first-order CT texture analysis. Acad Radiol. (2017) 24:1604–11. doi: 10.1016/j.acra.2017.07.008 28844845

[36] Yang Y Xu J Wang W Ma M Huang Q Zhou C. A nomogram based on the quantitative and qualitative features of CT imaging for the prediction of the invasiveness of ground glass nodules in lung adenocarcinoma. BMC Cancer. (2024) 24:438. doi: 10.1186/s12885-024-12207-8 38594670 PMC11005224

[37] Kwok WC Ma TF Ho JCM Lam DCL Sit KY Ip MSM. Prediction model on disease recurrence for low risk resected stage I lung adenocarcinoma. Respirology. (2023) 28:669–76. doi: 10.1111/resp.14508 37106570

[38] Shi L Shi W Peng X Zhan Y Zhou L Wang Y. Development and validation a nomogram incorporating CT radiomics signatures and radiological features for differentiating invasive adenocarcinoma from adenocarcinoma *in situ* and minimally invasive adenocarcinoma presenting as ground-glass nodules measuring 5-10mm in diameter. Front Oncol. (2021) 11:618677. doi: 10.3389/fonc.2021.618677 33968722 PMC8096901

[39] Park JE Kim D Kim HS Park SY Kim JY Cho SJ. Quality of science and reporting of radiomics in oncologic studies: room for improvement according to radiomics quality score and TRIPOD statement. Eur Radiol. (2020) 30:523–36. doi: 10.1007/s00330-019-06360-z 31350588

[40] Yanagawa M Ito R Nozaki T Fujioka T Yamada A Fujita S. New trend in artificial intelligence-based assistive technology for thoracic imaging. Radiol Med. (2023) 128:1236–49. doi: 10.1007/s11547-023-01691-w PMC10547663 37639191

