# Supplementary material for: CT morphological features and histogram parameters to predict micropapillary or solid components in stage IA lung adenocarcinoma
Source: Front Oncol. 2024 Jul 24;14:1448333. doi: 10.3389/fonc.2024.1448333 (PMC11303219; doi:10.3389/fonc.2024.1448333)
Supplement: Supplementary file 1 [file DataSheet_1.docx]

**1.** ***Tables for the construction of prediction models for MIP/SOL components in lung adenocarcinoma with tumor diameter less than 3cm.***

**Suppl Table 1:** Clinical characteristics and imaging information of the training and test sets for tumors ≤ 3cm.

|  | Overall（n＝376） | Train set（n＝264） | test set（n＝112） | *P* value |
| --- | --- | --- | --- | --- |
| **Pathological grouping, n (%)** |  |  |  | 0.298^#^ |
| MIP/SOL- | 282 (75.0) | 202 (76.5) | 80 (71.4) |  |
| MIP/SOL+ | 94 (25.0) | 62 (23.5) | 32 (28.6) |  |
| **Sex, n (%)** |  |  |  | 0.546^#^ |
| Male | 149 (39.6) | 102 (38.6) | 47 (42.0) |  |
| Female | 227 (60.4) | 162 (61.4) | 65 (58.0) |  |
| Age, years old | 62.00 [54.00, 67.00] | 62.00 [53.00, 67.00] | 63.00 [54.75, 68.00] | 0.288^*^ |
| **GGO, n (%)** |  |  |  | 0.100^#^ |
| solid nodule | 93 (24.7) | 59 (22.3) | 34 (30.4) |  |
| Nodules containing ground glass opacity | 283 (75.3) | 205 (77.7) | 78 (69.6) |  |
| **Lobulation, n (%)** |  |  |  | 0.914^#^ |
| No | 119 (31.6) | 84 (31.8) | 35 (31.2) |  |
| Yes | 257 (68.4) | 180 (68.2) | 77 (68.8) |  |
| **Spiculation, n (%)** |  |  |  | 0.122^#^ |
| No | 224 (59.6) | 164 (62.1) | 60 (53.6) |  |
| Yes | 152 (40.4) | 100 (37.9) | 52 (46.4) |  |
| **Vacuole sign, n (%)** |  |  |  | 0.071^#^ |
| No | 263 (69.9) | 192 (72.7) | 71 (63.4) |  |
| Yes | 113 (30.1) | 72 (27.3) | 41 (36.6) |  |
| **Pleural indentation, n (%)** |  |  |  | 0.417^#^ |
| No | 190 (50.5) | 137 (51.9) | 53 (47.3) |  |
| Yes | 186 (49.5) | 127 (48.1) | 59 (52.7) |  |
| **bronchial inflation sign, n (%)** |  |  |  | 0.899^#^ |
| No | 250 (66.5) | 175 (66.3) | 75 (67.0) |  |
| Yes | 126 (33.5) | 89 (33.7) | 37 (33.0) |  |
| **Vascular convergence, n (%)** |  |  |  | 0.179^#^ |
| No | 273 (72.6) | 197 (74.6) | 76 (67.9) |  |
| Yes | 103 (27.4) | 67 (25.4) | 36 (32.1) |  |
| **Location, n (%)** |  |  |  | 0.724^#^ |
| Left upper lobe | 97 (25.8) | 73 (27.7) | 24 (21.4) |  |
| Left lower lobe | 49 (13.0) | 34 (12.9) | 15 (13.4) |  |
| Right upper lobe | 137 (36.4) | 92 (34.8) | 45 (40.2) |  |
| Right middle lobe | 30 (8.0) | 22 (8.3) | 8 (7.1) |  |
| Right lower lobe | 63 (16.8) | 43 (16.3) | 20 (17.9) |  |
| **History of smoking, n (%)** |  |  |  | 0.373^#^ |
| No | 334 (88.8) | 237 (89.8) | 97 (86.6) |  |
| Yes | 42 (11.2) | 27 (10.2) | 15 (13.4) |  |
| **Family history of lung cancer, n (%)** |  |  |  | 0.881^△^ |
| No | 365(97.1) | 257 (97.3) | 108 (96.4) |  |
| Yes | 11 (2.9) | 7 (2.7) | 4(3.6) |  |
| **CEA, n (%)** |  |  |  | 0.622^#^ |
| Normal | 338 (89.9) | 236 (89.4) | 102 (91.1) |  |
| Rise | 38 (10.1) | 28 (10.6) | 10 (8.9) |  |
| **CYFRA21-1, n (%)** |  |  |  | 0.685^#^ |
| Normal | 311 (82.7) | 217 (82.2) | 94 (83.9) |  |
| Rises | 65 (17.3) | 47 (17.8) | 18 (16.1) |  |
| **NSE, n (%)** |  |  |  | 0.950^#^ |
| Normal | 261 (69.4) | 183 (69.3) | 78 (69.6) |  |
| Rise | 115 (30.6) | 81 (30.7) | 34 (30.4) |  |
| **PROGRP, n (%)** |  |  |  | 0.372^#^ |
| Normal | 349 (92.8) | 243 (92.0) | 106 (94.6) |  |
| Rise | 27 (7.2) | 21 (8.0) | 6 (5.4) |  |
| CTR | 32.25 [12.25, 60.60] | 31.90 [11.75, 60.05] | 32.90 [15.20, 64.90] | 0.560^*^ |
| Max slice area (mm^2^) | 131.41 [89.85, 205.38] | 134.87 [90.19, 215.93] | 128.64 [86.50, 182.23] | 0.339^*^ |
| 3D long axis (mm) | 15.75 [12.29, 19.47] | 16.01 [12.29, 19.82] | 15.35 [12.32, 18.03] | 0.338^*^ |
| Volume (mm^3^) | 1171.21 [630.95, 2189.20] | 1193.46 [634.86, 2300.70] | 1126.04[599.43, 1704.27] | 0.407^*^ |
| Nodule mass (mg) | 709.08 [353.57, 1491.32] | 707.91 [355.60, 1523.37] | 726.01 [351.98, 1395.77] | 0.977^*^ |
| Energy | 1.04 [0.46, 2.23] | 1.04 [0.44, 2.11] | 1.04 [0.50, 2.28] | 0.779^*^ |
| Entropy | 7.61 [6.95, 8.23] | 7.63 [6.95, 8.28] | 7.46 [6.95, 8.08] | 0.402^*^ |
| Kurtosis | 0.01 [-0.57, 1.08] | -0.04 [-0.59, 0.98] | 0.17 [-0.51, 1.39] | 0.220^*^ |
| Maximum CT value (Hu) | 161.00 [34.00, 508.00] | 176.50 [34.00, 509.00] | 129.50 [39.25, 494.00] | 0.830^*^ |
| Median CT value (Hu) | -448.40 [-558.60, -257.90] | -455.90 [-562.30, -306.03] | -388.65 [-556.18, -159.45] | 0.072^*^ |
| Average CT value (Hu) | -410.50 [-573.25, -188.00] | -424.50 [-574.00, -210.50] | -310.00 [-563.00, -59.25] | 0.087^*^ |
| Minimum CT value (Hu) | -901.50 [-1024.00, -767.75] | -913.00 [-1024.00, -769.75] | -878.00 [-1022.00, -733.50] | 0.111^*^ |
| Skewness | 0.22 [-0.36, 0.70] | 0.29 [-0.27, 0.71] | 0.09 [-0.58, 0.61] | 0.123^*^ |
| Standard deviation | 185.11 [149.26, 240.44] | 188.61 [149.38, 247.70] | 172.53 [148.25, 225.24] | 0.177^*^ |
| Sphericity | 0.78 [0.74, 0.81] | 0.77 [0.74, 0.81] | 0.78 [0.75, 0.81] | 0.634^*^ |
| Compactness | 0.47 [0.41, 0.54] | 0.46 [0.41, 0.54] | 0.47 [0.42, 0.54] | 0.634^*^ |

CEA, Carcinoembryonic antigen; CYFRA21-1, Cytokeratin 19; NSE, Neuron specific enolase; ProGRP, Pro-gastrin-releasing peptide.

# Pearsonχ^2^ test；△ continuous adjusted χ^2^ test；* Mann-Whitney U test.

**2.** ***Figures and tables for the construction of prediction models for MIP/SOL components in lung adenocarcinoma with tumor diameter less than 2cm.***

**Suppl Table 2:** Clinical characteristics and imaging information of the training and test sets for tumors ≤ 2cm.

|  | Overall（n＝293） | Train set（n＝208） | test set（n＝85） | *P* value |
| --- | --- | --- | --- | --- |
| **Pathological grouping, n (%)** |  |  |  | 0.088 ^#^ |
| MIP/SOL- | 226 (77.1) | 166 (79.8) | 60 (70.6) |  |
| MIP/SOL+ | 67 (22.9) | 42 (20.2) | 25 (29.4) |  |
| **Sex, n (%)** |  |  |  | 0.416 ^#^ |
| Male | 107 (36.5) | 79 (38.0) | 28 (32.9) |  |
| Female | 186 (63.5) | 129 (62.0) | 57 (67.1) |  |
| Age, years old | 61.00 [53.00, 67.00] | 60.00 [53.00, 66.25] | 62.00 [53.00, 67.00] | 0.534 ^*^ |
| **GGO, n (%)** |  |  |  | 0.934 ^#^ |
| solid nodule | 68 (23.2) | 48 (23.1) | 20 (23.5) |  |
| Nodules containing ground glass opacity | 225 (76.8) | 160 (76.9) | 65 (76.5) |  |
| **Lobulation, n (%)** |  |  |  | 0.060 ^#^ |
| No | 107 (36.5) | 83 (39.9) | 24 (28.2) |  |
| Yes | 186 (63.5) | 125 (60.1) | 61 (71.8) |  |
| **Spiculation, n (%)** |  |  |  | 0.600 ^#^ |
| No | 186 (63.5) | 134 (64.4) | 52 (61.2) |  |
| Yes | 107 (36.5) | 74 (35.6) | 33 (38.8) |  |
| **Vacuole sign, n (%)** |  |  |  | 0.981 ^#^ |
| No | 214 (73.0) | 152 (73.1) | 62 (72.9) |  |
| Yes | 79 (27.0) | 56 (26.9) | 23 (27.1) |  |
| **Pleural indentation, n (%)** |  |  |  | 0.195 ^#^ |
| No | 162 (55.3) | 110 (52.9) | 52 (61.2) |  |
| Yes | 131 (44.7) | 98 (47.1) | 33 (38.8) |  |
| **bronchial inflation sign, n (%)** |  |  |  | 0.914 ^#^ |
| No | 215 (73.4) | 153 (73.6) | 62 (72.9) |  |
| Yes | 78 (26.6) | 55 (26.4) | 23 (27.1) |  |
| **Vascular convergence, n (%)** |  |  |  | 0.863 ^#^ |
| No | 226 (77.1) | 161 (77.4) | 65 (76.5) |  |
| Yes | 67 (22.9) | 47 (22.6) | 20 (23.5) |  |
| **Location, n (%)** |  |  |  | 0.222 ^#^ |
| Left upper lobe | 75 (25.6) | 54 (26.0) | 21 (24.7) |  |
| Left lower lobe | 36 (12.3) | 21 (10.1) | 15 (17.6) |  |
| Right upper lobe | 108 (36.9) | 83 (39.9) | 25 (29.4) |  |
| Right middle lobe | 23 ( 7.8) | 17 ( 8.2) | 6 ( 7.1) |  |
| Right lower lobe | 51 (17.4) | 33 (15.9) | 18 (21.2) |  |
| **History of smoking, n (%)** |  |  |  | 0.941 ^#^ |
| No | 266 (90.8) | 189 (90.9) | 77 (90.6) |  |
| Yes | 27 ( 9.2) | 19 ( 9.1) | 8 ( 9.4) |  |
| **Family history of lung cancer, n (%)** |  |  |  | ＞0.999^☆^ |
| No | 290 (99.0) | 206 (99.0) | 84 (98.8) |  |
| Yes | 3 ( 1.0) | 2 ( 1.0) | 1 ( 1.2) |  |
| **CEA, n (%)** |  |  |  | 0.058 ^#^ |
| Normal | 269 (91.8) | 195 (93.8) | 74 (87.1) |  |
| Rise | 24 ( 8.2) | 13 ( 6.2) | 11 (12.9) |  |
| **CYFRA21-1, n (%)** |  |  |  | 0.277 ^#^ |
| Normal | 242 (82.6) | 175 (84.1) | 67 (78.8) |  |
| Rises | 51 (17.4) | 33 (15.9) | 18 (21.2) |  |
| **NSE, n (%)** |  |  |  | 0.316 ^#^ |
| Normal | 202 (68.9) | 147 (70.7) | 55 (64.7) |  |
| Rise | 91 (31.1) | 61 (29.3) | 30 (35.3) |  |
| **PROGRP, n (%)** |  |  |  | 0.852 ^#^ |
| Normal | 271 (92.5) | 192 (92.3) | 79 (92.9) |  |
| Rise | 22 ( 7.5) | 16 ( 7.7) | 6 ( 7.1) |  |
| CTR | 30.40 [10.30, 57.70] | 30.85 [11.55, 58.03] | 28.10 [8.70, 55.50] | 0.991 ^*^ |
| Max slice area (mm^2^) | 111.68 [75.70, 156.26] | 111.65 [79.32, 157.50] | 115.95 [73.72, 152.45] | 0.687 ^*^ |
| 3D long axis (mm) | 14.27 [11.50, 16.97] | 14.26 [11.52, 17.02] | 14.38 [11.41, 16.62] | 0.838 ^*^ |
| Volume (mm^3^) | 922.05 [537.40, 1440.42] | 906.72 [541.36, 1476.54] | 974.42 [527.62, 1315.41] | 0.958 ^*^ |
| Nodule mass (mg) | 569.22 [297.25, 903.68] | 555.30 [300.40, 868.26] | 636.84 [297.25, 934.56] | 0.619 ^*^ |
| Energy | 0.75 [0.38, 1.36] | 0.69 [0.38, 1.31] | 0.78 [0.39, 1.52] | 0.531 ^*^ |
| Entropy | 7.39 [6.82, 7.83] | 7.38 [6.83, 7.84] | 7.40 [6.82, 7.79] | 0.882 ^*^ |
| Kurtosis | 0.01 [-0.57, 0.98] | -0.01 [-0.57, 0.98] | 0.09 [-0.55, 0.92] | 0.626 ^*^ |
| Maximum CT value (Hu) | 219.00 [10.00, 508.00] | 261.00 [29.25, 503.00] | 104.00 [-6.00, 539.00] | 0.402 ^*^ |
| Median CT value (Hu) | -452.10 [-564.40, -278.20] | -448.40 [-560.98, -273.40] | -462.60 [-564.40, -313.30] | 0.644 ^*^ |
| Average CT value (Hu) | -425.00 [-574.00, -193.00] | -433.50 [-574.00, -198.50] | -370.00 [-574.00, -170.00] | 0.628 ^*^ |
| Minimum CT value (Hu) | -881.00 [-1024.00, -747.00] | -894.50 [-1024.00, -761.75] | -846.00 [-1005.00, -718.00] | 0.086 ^*^ |
| Skewness | 0.24 [-0.26, 0.71] | 0.25 [-0.26, 0.75] | 0.22 [-0.32, 0.67] | 0.666 ^*^ |
| Standard deviation | 180.55 [145.49, 242.24] | 187.53 [149.26, 245.72] | 162.10 [142.05, 226.86] | 0.080 ^*^ |
| Sphericity | 0.78 [0.75, 0.82] | 0.79 [0.75, 0.82] | 0.78 [0.74, 0.82] | 0.489 ^*^ |
| Compactness | 0.48 [0.42, 0.55] | 0.48 [0.43, 0.55] | 0.47 [0.41, 0.55] | 0.489 ^*^ |

CEA, Carcinoembryonic antigen; CYFRA21-1, Cytokeratin 19; NSE, Neuron specific enolase; ProGRP, Pro-gastrin-releasing peptide.

# Pearsonχ^2^ test；☆ Fisher's exact test；* Mann-Whitney U test.

**Suppl Table 3:** Comparison of clinical data indicators between the two groups in the training set for tumors ≤ 2cm.

|  | MIP/SOL- (n=166) | MIP/SOL+ (n=42) | *P* value |
| --- | --- | --- | --- |
| Age, years old | 60.00 [53.00, 67.00] | 60.00 [52.25, 64.75] | 0.548^*^ |
| Sex, n (%) |  |  | 0.466^#^ |
| Female | 61 (36.7) | 18 ( 42.9) |  |
| Male | 105 (63.3) | 24 ( 57.1) |  |
| History of smoking, n (%) |  |  | ＞0.999^△^ |
| No | 151 (91.0) | 38 ( 90.5) |  |
| Yes | 15 ( 9.0) | 4 ( 9.5) |  |
| Family history of lung cancer, n (%) |  |  | ＞0.999^☆^ |
| No | 164 (98.8) | 42 (100.0) |  |
| Yes | 2 ( 1.2) | 0 ( 0.0) |  |
| CEA, n (%) |  |  | 0.532^△^ |
| Normal | 157 (94.6) | 38 ( 90.5) |  |
| Rise | 9 ( 5.4) | 4 ( 9.5) |  |
| CYFRA21-1, n (%) |  |  | 0.432^#^ |
| Normal | 138 (83.1) | 37 ( 88.1) |  |
| Rise | 28 (16.9) | 5 ( 11.9) |  |
| NSE, n (%) |  |  | 0.309^#^ |
| Normal | 120 (72.3) | 27 ( 64.3) |  |
| Rise | 46 (27.7) | 15 ( 35.7) |  |
| ProGRP, n (%) |  |  | 0.411^△^ |
| Normal | 155 (93.4) | 37 ( 88.1) |  |
| Rise | 11 ( 6.6) | 5 ( 11.9) |  |

CEA,Carcinoembryonic antigen; CYFRA21-1,Cytokeratin 19; MIP, micropapillary; NSE,Neuron specific enolase; ProGRP,Pro-gastrin-releasing peptide; SOL,solid.

# Pearsonχ^2^ test；△ continuous adjusted χ^2^ test；☆ Fisher's exact test；* Mann-Whitney U test.


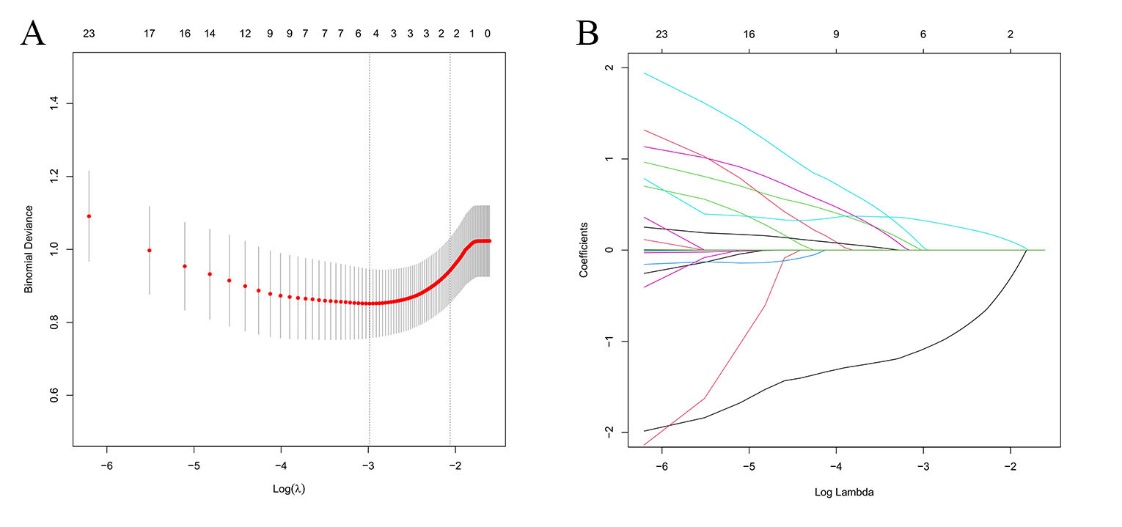


**Suppl Figure 1:** Lasso regression analysis diagram for tumors ≤ 2cm in the training set. **A**: Coefficient path of Lasso regression. **B**: Lasso regression cross-validation results.

**Suppl Table 4:** Multivariate logistic regression analysis for predicting MIP/SOL components in tumors ≤ 2cm.

| Variable | *OR* | 95%*CI* | *P* value |
| --- | --- | --- | --- |
| GGO | 0.230 | 0.080-0.626 | 0.005 |
| Entropy | 1.992 | 1.184-3.504 | 0.012 |
| AverageCTvalue | 1.002 | 1.000-1.004 | 0.018 |

GGO, ground-glass opacity; CT, computed tomography.
